# Transmission Dynamics of COVID-19 Outbreaks Associated with Child Care Facilities — Salt Lake City, Utah, April–July 2020

**DOI:** 10.15585/mmwr.mm6937e3

**Published:** 2020-09-18

**Authors:** Adriana S. Lopez, Mary Hill, Jessica Antezano, Dede Vilven, Tyler Rutner, Linda Bogdanow, Carlene Claflin, Ian T. Kracalik, Victoria L. Fields, Angela Dunn, Jacqueline E. Tate, Hannah L. Kirking, Tair Kiphibane, Ilene Risk, Cuc H. Tran

**Affiliations:** ^1^CDC COVID-19 Response Team; ^2^Salt Lake County Health Department, Utah; ^3^Utah Department of Health.

*On September 11, 2020, this report was posted online as an *MMWR *Early Release.*

Reports suggest that children aged ≥10 years can efficiently transmit SARS-CoV-2, the virus that causes coronavirus disease 2019 (COVID-19) (*1*,*2*). However, limited data are available on SARS-CoV-2 transmission from young children, particularly in child care settings (*3*). To better understand transmission from young children, contact tracing data collected from three COVID-19 outbreaks in child care facilities in Salt Lake County, Utah, during April 1–July 10, 2020, were retrospectively reviewed to explore attack rates and transmission patterns. A total of 184 persons, including 110 (60%) children had a known epidemiologic link to one of these three facilities. Among these persons, 31 confirmed COVID-19 cases occurred; 13 (42%) in children. Among pediatric patients with facility-associated confirmed COVID-19, all had mild or no symptoms. Twelve children acquired COVID-19 in child care facilities. Transmission was documented from these children to at least 12 (26%) of 46 nonfacility contacts (confirmed or probable cases). One parent was hospitalized. Transmission was observed from two of three children with confirmed, asymptomatic COVID-19. Detailed contact tracing data show that children can play a role in transmission from child care settings to household contacts. Having SARS-CoV-2 testing available, timely results, and testing of contacts of persons with COVID-19 in child care settings regardless of symptoms can help prevent transmission. CDC guidance for child care programs recommends the use of face masks, particularly among staff members, especially when children are too young to wear masks, along with hand hygiene, frequent cleaning and disinfecting of high-touch surfaces, and staying home when ill to reduce SARS-CoV-2 transmission (*4*).

Contact tracing* data collected during April 1–July 10, 2020 through Utah’s National Electronic Disease Surveillance System (EpiTrax) were used to retrospectively construct transmission chains from reported COVID-19 child care facility outbreaks, defined as two or more laboratory-confirmed COVID-19 cases within 14 days among staff members or attendees at the same facility. EpiTrax maintains records of epidemiologic linkage between index patients and contacts (defined as anyone who was within 6 feet of a person with COVID-19 for at least 15 minutes ≤2 days before the patient’s symptom onset) and captures data on demographic characteristics, symptoms, exposures, testing, and the monitoring/isolation period. A confirmed case was defined as receipt of a positive SARS-CoV-2 real-time reverse transcription–polymerase chain reaction (RT-PCR) test result. A probable case was an illness with COVID-19–compatible symptoms,^†^ epidemiologically linked to the outbreak, but with no laboratory testing. For this report, the index case was defined as the first confirmed case identified in a person at the child care facility, and the primary case was defined as the earliest confirmed case linked to the outbreak. Pediatric patients were aged <18 years; adults were aged ≥18 years.

Persons with confirmed or probable child care facility–associated COVID-19 were required to isolate upon experiencing symptoms or receiving a positive SARS-CoV-2 test result. Contacts were required to quarantine for 14 days after contact with a person with a confirmed case. Facility attack rates were calculated by including patients with confirmed and probable facility-associated cases (including the index patient) in the numerator and all facility staff members and attendees in the denominator. Overall attack rates include facility-associated cases (including the index case) and nonfacility contact (household and nonhousehold) cases in the numerator and all facility staff members and attendees and nonfacility contacts in the denominator; the primary case and cases linked to the primary case are excluded.

During April 1–July 10, Salt Lake County identified 17 child care facilities (day care facilities and day camps for school-aged children; henceforth, facilities) with at least two confirmed COVID-19 cases within a 14-day period. This report describes outbreaks in three facilities that experienced possible transmission within the facility and had complete contact investigation information. A total of 184 persons, including 74 (40%) adults (median age = 30 years; range = 19–78 years) and 110 (60%) children (median age = 7 years; range = 0.2–16 years), had a known epidemiologic link to one of these three facilities with an outbreak; 54% were female and 40% were male. Among these persons, 31 confirmed COVID-19 cases occurred (Table 1); 18 (58%) cases occurred in adults and 13 (42%) in children. Among all contacts, nine confirmed and seven probable cases occurred; the remaining 146 contacts had either negative test results (50; 27%), were asymptomatic and were not tested (94; 51%) or had unknown symptoms and testing information (2; 1%).

**TABLE 1:** Characteristics of all staff members, attendees, and their contacts associated with COVID-19 outbreaks at three child care facilities — Salt Lake County, Utah, April 1–July 10, 2020

Characteristic	No. (% with available information)		
	Total*	Adult*	Pediatric*
**Facility staff members, attendees, and contacts**	184 (100)	74 (100)	110 (100)
**Age, yrs, median (range)^†^**	9 (0.2–78)	30 (19–78)	7 (0.2–16)
**Sex**			
Female	100 (54)	42 (57)	58 (53)
Male	74 (40)	31 (42)	43 (39)
Unavailable	10 (5)	1 (1)	9 (8)
**Linkage to facility**			
Facility staff member or attendee	101 (55)	18 (24)	83 (75)
Nonfacility contact^§^	83 (45)	56 (76)	27 (25)
**Confirmed^¶^ COVID-19 cases**			
Total	31 (17)	18 (24)	13 (12)
Symptomatic	24 (13)	15 (24)	9 (8)
Index case at facility	3 (2)	3 (4)	0 (–)
Asymptomatic	4 (2)	0 (–)	4 (4)
**Probable^¶^ COVID-19 cases**	7 (4)	5 (7)	2 (2)
**Contacts^§^**			
Total	146 (79)	51 (60)	95 (86)
Contacts with a negative test result	50 (27)	27 (36)	23 (21)
Asymptomatic contacts, not tested	94 (51)	22 (30)	72 (65)
Contacts with unknown symptoms and testing	2 (1)	2 (3)	0 (—)

**Abbreviation:** COVID-19 = coronavirus disease 2019.

* Does not include two persons with primary cases or their six contacts; two adult contacts had unknown symptom and testing information. Percent is calculated as a percentage of the total.

^†^ Age data were missing for 11 contacts.

^§^ Includes pediatric and adult household and nonhousehold contacts.

^¶^ A confirmed case was defined as a positive SARS-CoV-2 reverse transcription–polymerase chain reaction test result. A probable case was an illness with symptoms consistent with COVID-19 and linked to the outbreak but without laboratory testing.

Among the 101 facility staff members and attendees, 22 (22%) confirmed COVID-19 cases (10 adult and 12 pediatric) were identified (Table 2), accounting for 71% of the 31 confirmed cases; the remaining nine (29%) cases occurred in contacts of staff members or attendees. Among the 12 facility-associated pediatric patients with confirmed COVID-19, nine had mild symptoms, and three were asymptomatic. Among 83 contacts of these 12 pediatric patients, 46 (55%) were nonfacility contacts, including 12 (26%) who had confirmed (seven) and probable (five) COVID-19. Six of these cases occurred in mothers and three in siblings of the pediatric patients. Overall, 94 (58%) of 162 contacts of persons with facility-associated cases had no symptoms of COVID-19 and were not tested. Staff members at two of the facilities had a household contact with confirmed or probable COVID-19 and went to work while their household contact was symptomatic. These household contacts represented the primary cases in their respective outbreaks.

**TABLE 2:** Classification of contacts with known linkage to facility-associated confirmed adult and pediatric cases* at three child care facilities — Salt Lake County, Utah, April 1–July 10, 2020

Classification	No. (%)					
	Total^†^	Adult^†^	Pediatric	Facility		
				A	B	C
**COVID-19 cases at facilities^§^**	22	10	12	2	5	15
**Contacts**^¶^ **linked to cases at facilities**	162	79	83	25	28	109
Contacts^¶^ with confirmed COVID-19	9 (6)	2 (3)	7 (8)	0 (—)	4 (14)	5 (5)
Contacts^¶^ with probable COVID-19	7 (4)	2 (3)	5 (6)	0 (—)	3 (11)	4 (4)
Contacts^¶^ with negative test results	50 (31)	25 (32)	25 (30)	3 (12)	13 (46)	34 (31)
Asymptomatic contacts, not tested	94 (58)	48 (61)	46 (55)	20 (80)	8 (29)	66 (61)
Contacts with unknown symptoms and testing	2 (1)	2 (3)	0 (—)	2 (1)	0 (—)	0 (—)
**Interval (days)**						
Facility case onset to contact onset, median (range)**	4 (1–8)	6 (4–6)	3 (1–8)	1 (1–1)	4.5 (1–6)	4 (3–8)
Facility case onset to testing, median (range)^††^	2.5 (0–6)	1 (0–4)	4 (1–6)	2.5 (1–4)	1 (0–3)	2 (0–10)

**Abbreviation:** COVID-19 = coronavirus disease 2019.

* A confirmed case was defined as a positive SARS-CoV-2 reverse transcription–polymerase chain reaction test result. A probable case was an illness with symptoms consistent with COVID-19 and linked to the outbreak but without laboratory testing.

^†^ A positive adult case linked to facility attendee from Facility B is included because they were a staff member.

^§^ Includes index cases.

^¶^ Includes pediatric and adult household and nonhousehold contacts.

** For cases in persons who were asymptomatic, onset for contact is date of receipt of positive test result.

^††^ Does not include three pediatric facility cases in persons who were asymptomatic who did not have symptom onset dates.

## Facility A Outbreak

Facility A, which had been deemed an essential business and had not closed before the outbreak occurred, required daily temperature and symptom screening for the 12 staff members and children and more frequent cleaning and disinfection; staff members were required to wear masks. Two COVID-19 cases in staff members were associated with facility A (Figure). The index case at facility A (patient A1) occurred in a staff member who reported symptom onset on April 2, self-isolated on April 3, and had a positive SARS-CoV-2 RT-PCR test result from a nasopharyngeal (NP) swab specimen obtained on April 6. Three days after patient A1’s symptom onset, a second staff member (patient A2) experienced symptoms and had a positive SARS-CoV-2 test result 1 day later. Ten facility contacts (nine children aged 1–5 years and one staff member) remained asymptomatic during the monitoring period and were not tested. The last reported exposure at facility A was on April 3, when the facility closed. Among the 15 nonfacility contacts of patients A1 and A2 (including four children aged 1–13 years), 10 remained asymptomatic throughout their monitoring period and were not tested, and three received negative test results; the symptom and testing information for two nonfacility contacts was unknown. The primary patient, a household contact of the index patient, reported symptom onset 9 days before symptom onset in patient A1 and received a positive SARS-CoV-2 test result from an NP specimen collected on April 6. The facility attack rate (excluding the primary case) for facility A was 17% (two of 12) and was 7% overall (including contacts) (two of 27).

**FIGURE:**
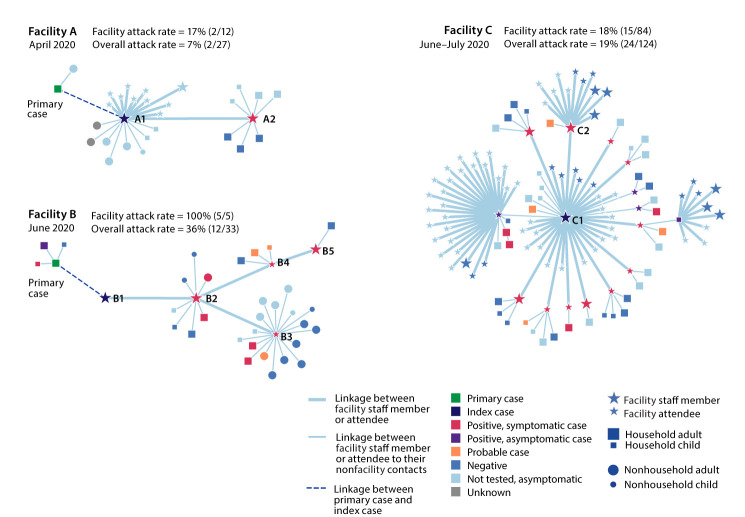
Transmission chains* and attack rates^†^^,^^§^ in three COVID-19 child care facility outbreaks^¶^^,^**^,††^ — Salt Lake County, Utah, April 1– July 10, 2020 **Abbreviation:** COVID-19 = coronavirus disease 2019. * Transmission chains developed using Microbe Trace software. https://www.biorxiv.org/content/10.1101/2020.07.22.216275v1. ^†^ Facility attack rates include index cases and all facility staff members and attendees. ^§^ Overall attack rates include all facility staff members and attendees (including the index case) and nonfacility contacts (household and nonhousehold). It does not include the primary case or the cases linked to the primary case. ^¶^ A confirmed case was defined as a positive SARS-CoV-2 reverse transcription–polymerase chain reaction test result. A probable case was an illness with symptoms consistent with COVID-19 and linked to the outbreak but without laboratory testing. ** The index case was defined as the earliest confirmed case in a person at the child care facility. ^††^ A primary case was defined as the earliest confirmed case linked to the outbreak.

## Facility B Outbreak

Facility B was closed during March 13–May 4. Upon reopening, temperatures of the five staff members and children were checked daily, and more frequent cleaning was conducted; only staff members were required to wear masks. Five COVID-19 cases in three staff members and two children were associated with facility B (Figure). The index case (B1) occurred in a staff member who was tested on May 31 while presymptomatic (because of a household contact with COVID-19) and received a SARS-CoV-2-positive test result; patient B1 experienced mild COVID-19 symptoms on June 3 and last worked on May 29. A second staff member (patient B2), experienced symptoms on June 8, was tested, and received a positive test result 2 days later. Patients B3 and B4, children aged 8 months and 8 years, respectively, experienced mild signs and symptoms (fever, fatigue, runny nose) 7 and 8 days, respectively, after symptom onset in patient B2; both children were tested and received positive test results the day after their symptoms commenced. A third staff member, patient B5, experienced symptoms 9 days after symptoms occurred in patient B4, was tested, and received a positive test result 1 day later. The two children likely transmitted SARS-CoV-2 to their contacts including two confirmed cases (in one child’s mother and father, both symptomatic 2 and 3 days, respectively, following the child’s illness onset) and three probable cases (in two adults, including one mother and a child). The index patient (B1) was a household contact of the primary patient who had symptom onset May 26, was tested on May 29, and received a positive SARS-CoV-2 test result. The facility attack rate for facility B was 100% (five of five) and the overall attack rate was 36% (12 of 33).

## Facility C Outbreak

Facility C was closed during March 13–June 17. Upon reopening, the facility requested that 84 staff members and children check their temperature and monitor their symptoms daily; masks were not required for staff members or children. Fifteen COVID-19 cases (in five staff members and 10 children) were associated with facility C (Figure). Two staff members and two students reported symptoms on June 24 and self-isolated. The index case occurred in a staff member (patient C1), who had a positive test result from an NP specimen obtained on June 25. The second staff member, patient C2, was tested 2 days later and received a positive SARS-CoV-2 test result, and the two students (aged 7 and 8 years) were tested on June 28 and 29, respectively and received positive test results. Over the subsequent 8 days, an additional eight students (aged 6–10 years), three of whom were asymptomatic, and three staff members (all symptomatic) received positive SARS-CoV-2 test results. Pediatric patients at the facility likely transmitted SARS-CoV-2 to their contacts, including five confirmed cases in household contacts (three mothers, one aunt, and one child) and two probable household cases (one mother and one child). Symptoms developed 3 and 5 days following the child’s illness onset when onset date was known. One mother who was presumably infected by her asymptomatic child was subsequently hospitalized. Among the seven cases in symptomatic children, fever was the most common sign, followed by symptoms of headache and sore throat. The source for this cluster was not identified. The facility attack rate for facility C was 18% (15 of 84) and the overall attack rate was 19% (24 of 124).

## Discussion

Analysis of contact tracing data in Salt Lake County, Utah, identified outbreaks of COVID-19 in three small to large child care facilities linked to index cases in adults and associated with transmission from children to household and nonhousehold contacts. In these three outbreaks, 54% of the cases linked to the facilities occurred in children. Transmission likely occurred from children with confirmed COVID-19 in a child care facility to 25% of their nonfacility contacts.

Mitigation strategies^§^ could have helped limit SARS-CoV-2 transmission in these facilities. To help control the spread of COVID-19, the use of masks is recommended for persons aged ≥2 years.^¶^ Although masks likely reduce the transmission risk (*5*), some children are too young to wear masks but can transmit SARS-CoV-2, as was seen in facility B when a child aged 8 months transmitted SARS-CoV-2 to both parents.

The findings in the report are subject to at least three limitations. First, guidance for contact tracing methodology changed during the pandemic and could have resulted in differences in data collected over time. Second, testing criteria initially included only persons with typical COVID-19 signs and symptoms of fever, cough, and shortness of breath, which could have led to an underestimate of cases and transmission. Finally, because the source for the outbreak at facility C was unknown, it is possible that cases associated with facility C resulted from transmission outside the facility.

COVID-19 is less severe in children than it is in adults (*6*,*7*), but children can still play a role in transmission (*8*,*9*). The infected children exposed at these three facilities had mild to no symptoms. Two of three asymptomatic children likely transmitted SARS-CoV-2 to their parents and possibly to their teachers. Having SARS-CoV-2 testing available, timely results, and testing of contacts of patients in child care settings regardless of symptoms can help prevent transmission and provide a better understanding of the role played by children in transmission. Findings that staff members worked while their household contacts were ill with COVID-19–compatible symptoms support CDC guidance for child care programs recommendations that staff members and attendees quarantine and seek testing if household members are symptomatic (*4*). This guidance also recommends the use of face masks, particularly among staff members, especially when children are too young to wear masks, along with hand hygiene, frequent cleaning and disinfecting of high-touch surfaces, and staying home when ill to reduce SARS-CoV-2 transmission.

**Summary**

**What is already known about this topic?**

Children aged ≥10 years have been shown to transmit SARS-CoV-2 in school settings.

**What is added by this report?**

Twelve children acquired COVID-19 in child care facilities. Transmission was documented from these children to at least 12 (26%) of 46 nonfacility contacts (confirmed or probable cases). One parent was hospitalized. Transmission was observed from two of three children with confirmed, asymptomatic COVID-19.

**What are the implications for public health practice?**

SARS-CoV-2 Infections among young children acquired in child care settings were transmitted to their household members. Testing of contacts of laboratory-confirmed COVID-19 cases in child care settings, including children who might not have symptoms, could improve control of transmission from child care attendees to family members.

## Footnotes

* https://www.cdc.gov/coronavirus/2019-ncov/php/contact-tracing/contact-tracing-plan/contact-tracing.html.

† https://www.cdc.gov/coronavirus/2019-ncov/symptoms-testing/symptoms.html.

§ https://www.cdc.gov/coronavirus/2019-ncov/if-you-are-sick/isolation.html; https://www.cdc.gov/coronavirus/2019-ncov/if-you-are-sick/quarantine.html.

¶ https://www.cdc.gov/coronavirus/2019-ncov/prevent-getting-sick/cloth-face-cover-guidance.html.
